# Production of Factor VIII by Human Liver Sinusoidal Endothelial Cells Transplanted in Immunodeficient uPA Mice

**DOI:** 10.1371/journal.pone.0077255

**Published:** 2013-10-22

**Authors:** Marina E. Fomin, Yanchen Zhou, Ashley I. Beyer, Jean Publicover, Jody L. Baron, Marcus O. Muench

**Affiliations:** 1 Blood Systems Research Institute, San Francisco, California, United States of America; 2 Department of Laboratory Medicine, University of California San Francisco, San Francisco, California, United States of America; 3 Department of Medicine, University of California San Francisco, San Francisco, California, United States of America; 4 Liver Center, University of California San Francisco, San Francisco, California, United States of America; Institut National de la Santé et de la Recherche Médicale, France

## Abstract

Liver sinusoidal endothelial cells (LSECs) form a semi-permeable barrier between parenchymal hepatocytes and the blood. LSECs participate in liver metabolism, clearance of pathological agents, immunological responses, architectural maintenance of the liver and synthesis of growth factors and cytokines. LSECs also play an important role in coagulation through the synthesis of Factor VIII (FVIII). Herein, we phenotypically define human LSECs isolated from fetal liver using flow cytometry and immunofluorescence microscopy. Isolated LSECs were cultured and shown to express endothelial markers and markers specific for the LSEC lineage. LSECs were also shown to engraft the liver when human fetal liver cells were transplanted into immunodeficient mice with liver specific expression of the urokinase-type plasminogen activator (uPA) transgene (uPA-NOG mice). Engrafted cells expressed human Factor VIII at levels approaching those found in human plasma. We also demonstrate engraftment of adult LSECs, as well as hepatocytes, transplanted into uPA-NOG mice. We propose that overexpression of uPA provides beneficial conditions for LSEC engraftment due to elevated expression of the angiogenic cytokine, vascular endothelial growth factor. This work provides a detailed characterization of human midgestation LSECs, thereby providing the means for their purification and culture based on their expression of CD14 and CD32 as well as a lack of CD45 expression. The uPA-NOG mouse is shown to be a permissive host for human LSECs and adult hepatocytes, but not fetal hepatoblasts. Thus, these mice provide a useful model system to study these cell types in vivo. Demonstration of human FVIII production by transplanted LSECs encourages further pursuit of LSEC transplantation as a cellular therapy for the treatment of hemophilia A.

## Introduction

Liver sinusoidal endothelial cells (LSECs) form a layer partially separating hepatocytes from the blood in liver sinusoids. LSECs play a number of important roles including blood filtration, cytokine secretion and immune regulation [1]–[3]. LSECs differ from vascular endothelial cells in that they lack a basal membrane and have large pores called fenestrae. Fenestrae allow passage of plasma and particles between blood and the space of Disse, thus allowing hepatocytes to perform their metabolic functions [4]. LSECs originate during embryogenesis from the capillary endothelium of the septum transversum [5]. Between 5 and 20 weeks’ gestation LSECs lose their basal membranes, acquire fenestrations and start production of extracellular matrix components.

Functional differences between LSECs and vascular endothelial cells are further reflected in the proteome. There are, however, inconsistencies in the published phenotypes of LSECs [1]. For instance, some researchers describe LSECs as CD31^−^ (platelet endothelial cell adhesion molecule-1^−^), CD34^−^ and von Willebrand Factor (vWF)^−^ [5]–[7], whereas others have observed expression of these markers on LSECs [1], [8], [9]. Difficulties in obtaining sufficient numbers of freshly isolated human LSECs for study has in some cases led to the reliance on culture expanded LSECs. As in vitro culture generally has strong affects on gene expression, the phenotypic profile of cultured cells is likely to differ from that of LSECs in their natural state. Differences between mouse and human LSECs in cell-surface expression of immune-modulatory factors have also been suggested [1], [10]. With these points in mind, we endeavored to profile the expression of cell-surface antigens on freshly-isolated LSECs obtained from fetal human livers.

One of the many important functions of LSECs is production of Factor VIII (FVIII) [11]. FVIII is a blood coagulation factor, the deficit of which causes hemophilia A, a lethal disease without treatment. Therapeutic administration of recombinant FVIII prevents fatal bleeding. Transplantation of LSECs could provide a permanent source of FVIII and a cure for hemophilia A. Successful transplantation of rodent LSECs has been described in the CD26 (dipeptidyl peptidase IV, DPPIV) knockout rat [12], [13] and in a hemophilia A mouse model [9], [14]. Benten et al. demonstrated a dispersed distribution of LSECs shortly after transplant into mice with long-term survival in the liver [8]. Transplantation of human LSECs with production FVIII has not been described.

We report transplantation of human liver cells into NOD.Cg-*Prkdc^scid^ Il2 rg^tm1Sug^ Tg(Alb-Plau)11-4*/ShiJic (uPA-NOG) mice. These are immunodeficient mice, which have a mouse urokinase-type plasminogen activator (uPA) transgene expressed under a murine albumin promoter [15]. This mouse has been successfully transplanted with human adult hepatocytes. In this study, we sought to evaluate liver engraftment in uPA-NOG mice transplanted with human fetal liver cells and adult liver cells, for comparison. Our findings indicate that LSECs readily engraft the livers of uPA-NOG mice and produce FVIII that can be measured in the plasma.

## Materials and Methods

### Ethics Statement

Human fetal tissues were obtained from elective abortions with the approval of the Committee for Human Research at the University of California San Francisco and with written consent of the women undergoing the abortion. All specimens were anonymous.

Animal research was performed with approval of the Institutional Animal Care and Use Committee at ISIS Services LLC (San Carlos, CA, USA). Mice received humane care according to the criteria outlined by the National Research Council’s Institute of Laboratory Animal Resources in the “Guide for the Care and Use of Laboratory Animals”.

### Human Tissues and Cell Isolation

The gestational age of the liver specimens was estimated based on the foot-length of the fetus. Midgestation specimens up to 24 weeks of age were obtained for this study. Procurement, transport and cell isolation from fetal livers was performed as previously described [16]. Briefly, tissue were homogenized and enzymatically digested with 1.0 mg/mL collagenase blend Liberase Cl or, in later experiments, Liberase T-Flex and 0.005% DNase (Roche Diagnostic, Indianapolis, IN). Red cells were depleted with monoclonal CD235 antibody using immunomagnetic beads. Cell sorting was performed on FACSAria flow cytometer (BD, Franklin Lakes, NJ) [16].

Frozen adult-human hepatocytes preparations were obtained from Lonza (Lonza, Walkersville, MD).

### Flow Cytometry Analysis

Cells were stained with monoclonal antibodies (see Table S1) and analyzed on LSR II flow cytometer (BD). Analyses of flow cytometric data focused only on live cells using an electronic gate to identify propidium iodide (PI) ^−^ events using FlowJo software (Tree Star, Inc., Ashland, OR).

### Quantitative Real-time Reverse-transcriptase Polymerase Chain Reaction (qPCR) Analysis

qPCR analysis methods were described in [16]. Primers used in this study are listed in Table S2.

### Cell Culture

Cells were cultured in EGM-2 BulletKit supplemented medium (Lonza) in 8-well collagen-coated Biocoat® slides (BD) for 3 weeks and then fixed for immunofluorescence staining. Fresh medium was added weekly.

### Immunofluorescence Staining for Epifluorescence Microscopy

Pieces of tissue were fixed and embedded in optimal cutting temperature compound (OCT; Tissue-Tek®) as described previously [16]. 10 µm cryostat sections were incubated with primary antibodies, washed and incubated with corresponding secondary antibodies (Table S1). Slides were covered with ProLong® Gold antifade reagent with 40′,6-diamidino-2-phenylindole (DAPI) (Life Technologies, Grand Island, NY). Images were analyzed with a Leica CTR6500 (Leica Microsystems, Buffalo Grove, IL). Colocalization analysis was performed using iVision software (BioVision Technologies, Exton, PA).

### Experimental Mice

Mice were maintained in a restricted access, specific-pathogen free vivarium at Blood Systems Research Institute as previously detailed [17]. Founder NOD.Cg-Prkdc^scid^ Il2**rg^tm1Wjl^/SzJ (NSG) mice were obtained from Jackson Laboratories (Sacramento, CA, USA). Founder uPA-NOG mice were obtained from the Central Institute for Experimental Animals (Kawasaki, Japan) [15]. The line of mice was maintained through pairings of male homozygous and female hemizygous mice. The levels of alanine aminotransferase were measured in the serum of offspring mice by using an ALT-L3K kit (Sekisui Diagnostics P.E.I Inc., Charlottetown, PE, Canada) on a Cobas Miras Plus analyzer (Roche Diagnostics) at 8 weeks’ age and mice with levels >100 U/L were considered to be homozygous for the uPA transgene.

### Transplantation of Human Liver Cells and Analysis of Engraftment

All surgeries were performed using inhalation anesthesia and mice were given food-borne analgesic medication following their procedures to minimize pain and suffering. Fetal or adult human cells were transplanted intrasplenically into homozygous uPA-NOG mice 8 to 10 weeks old (Table 1). Mice were sacrificed 100 to 233 days after transplantation and livers, spleens, bone marrow and blood harvested. Bone marrow and the light-density fraction of splenic cells were analyzed by flow cytometry [17]. Part of each liver was used for cryo-sectioning and immunofluorescent staining. The remaining portion of liver was digested with collagenase IV (Life Technologies), stained for mouse and human antigens and analyzed by flow cytometry.

**Table 1:** Summary of cell and recipient uPA-NOG mouse numbers used in individual transplant experiments.

Experiment Number	Gestational age of human liver (weeks)	Approximated cell dose*	Number of mice
1	23	0.5×10^6^	8
2	23	1×10^6^	7
3	21	2×10^6^	8
4	23	1.2×10^6^	10
5	21	1×10^6^	2
6	15	3×10^6^	10

* The actual amount of cells transplanted into each animal can in some cases be lower because of the sample leakage during injections.

### Human FVIII in Mouse Plasma Measurement

Plasma was separated from blood collected from transplanted mice. Human FVIII concentration was determined by ELISA using the Matched-Pair Antibody set for the human FVIII antigen following the manufacturer’s protocol (Affinity Biologicals, Ancaster, ON, Canada) with the following modifications: The ELISA signal was detected using SuperSignal ELISA Pico Chemiluminescent Substrate (Thermo Scientific, Rockford, IL) and luminescence measured using a POLARstar luminometer (BMG LABTECH Inc., Cary, NC). Standard curves were generated using VisuCal-F Frozen Calibrator plasma (Affinity Biologicals), representing a pool of human plasma collected from a minimum of 20 donors and FVIII activity was calibrated as 108% of the secondary coagulation standard lot #4 reference standard from the Scientific and Standardization Committee of the International Society of Thrombosis and Haemostasis. According to the lot #4 reference standard, the VisuCal-F Frozen Calibrator plasma is equivalent to 0.95 IU/ml FVIII. An additional human plasma sample, used in the assay as an independent positive control, was obtained from our institute and handled in a similar fashion as the murine plasma samples. Each plasma sample was diluted by four serial dilutions (4-, 8-, 16-, and 32-fold) with ELISA sample diluent and measured in triplicate. Data are shown as the undiluted mean percent-value of the lot #4 reference standard ± standard deviation from results obtained with samples diluted 8-fold.

### Mouse VEGF Measurement

Livers and serum were obtained from uPA-NOG homozygous, hemizygous and NSG mice (n = 9; 5 females and 4 males for each group). Mouse ages varied from 4 to 9 months in each group. 500 mg of each liver was homogenized in RIPA buffer (Thermo Scientific) with Proteinase Inhibitor cocktail (Roche Diagnostics). VEGF ELISA was performed with mouse VEGF polyclonal antibodies Duo-Set mouse VEGF (R&D Systems, Minneapolis, MN) according to the manufacturer’s instructions with the following modifications: The ELISA signal was detected using SuperSignal ELISA Pico Chemiluminescent Substrate (Thermo Scientific, Rockford, IL) and luminescence measured using a POLARstar luminometer (BMG LABTECH Inc., Cary, NC). Total protein concentration was measured with the bicinchoninic acid (BCA) protein assay kit (Pierce Biotechnology, Rockford, IL). Relative VEGF concentrations in livers were calculated as a ratio of VEGF concentration to total protein concentration.

### Statistical Analysis

The significance of differences in the levels of gene expression measured by qPCR was determined using the nonparametric two-tailed Mann-Whitney U test performed using Aabel 3 software (Gigawiz Ltd. Co. OK, USA). VEGF concentration data were analyzed with GraphPad Prism 5.0 software. Statistical analysis of values was performed using unpaired two-tailed Student’s *t*-test between groups and *P*≤0.05 was considered significant.

## Results

### Identification and Phenotypic Characterization of Human Fetal LSECs

**Figure 1:**
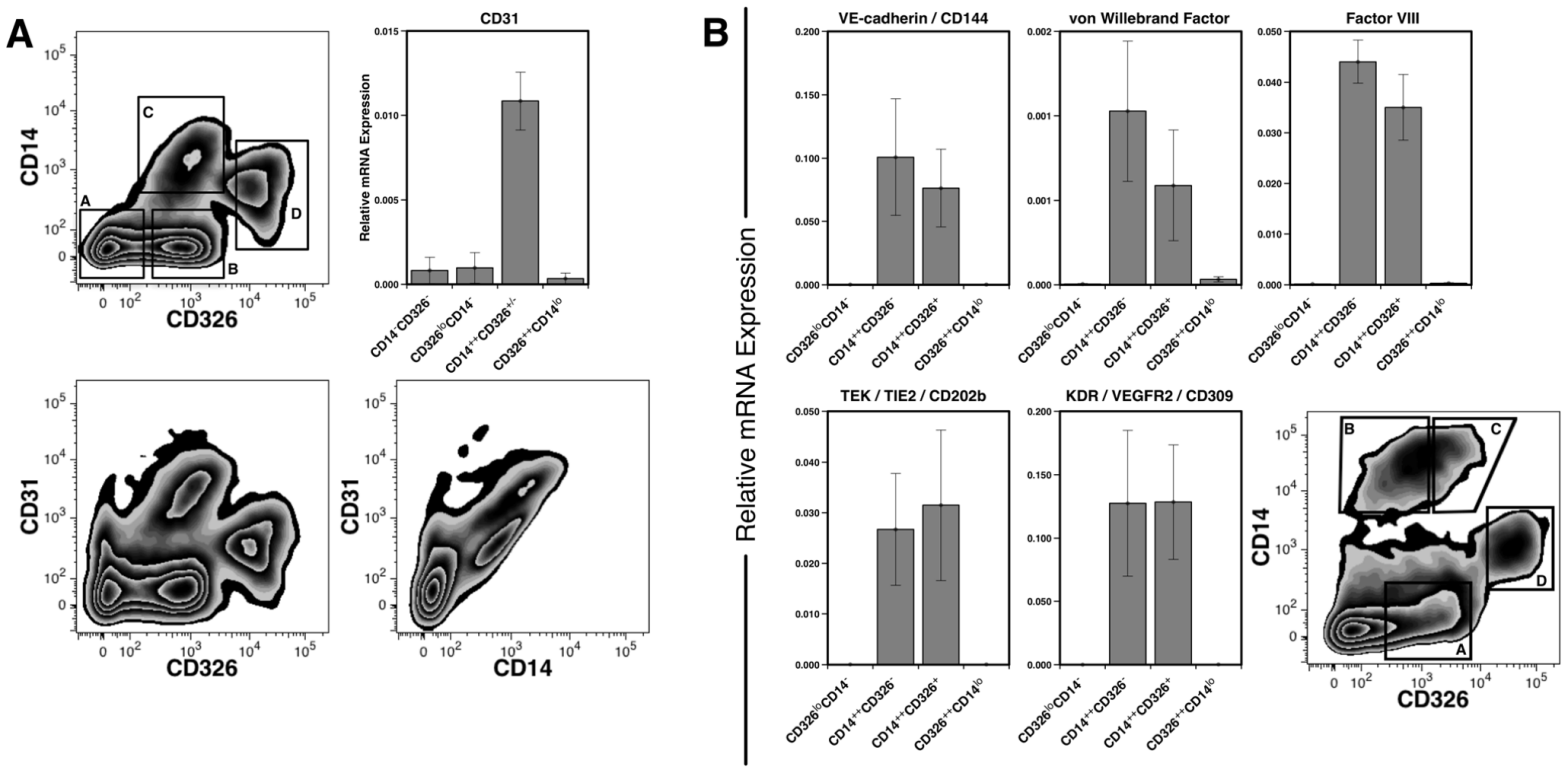
Gene expression by fetal LSECs. CD31 expression on CD14^++^ cells measured by flow cytometry and mRNA levels. (**A**). Viable non-hematopoietic cells (PI^−^CD45^−^) gated by CD326 and CD14 expression (a: CD14^−^CD326^−^; b: CD326^lo^CD14^−^; c: CD14^++^CD326^+/−^; d: CD326^++^CD14^lo^). Expression of CD31 was analyzed by qPCR in each sorted population (n = 3). Flow cytometric plots shows expression of CD31 versus CD326 and CD31 versus CD14. (**B**): Gene expression profile of sorted populations (a: CD326^lo^CD14^−^; b: CD14^++^CD326^−^; c: CD14^++^CD326^+^; d: CD326^++^CD14^lo^) measured by qPCR, n = 7.

Previously we described a method for the discrimination of fetal liver cells based on the combined expression of the surface markers CD326 (epithelial cell adhesion molecule) and the lipopolysaccharide receptor CD14, and a lack of the blood cell marker CD45. Whereas cells expressing high levels of CD326 and low levels of CD14 represent hepatoblasts (Fig. 1A, region D) [16], another population of cells identified by this staining schema, which we refer to as CD14^++^ cells (Fig. 1A, region C), was found to express significantly more CD31 mRNA than cells found in groups B or D (*P*<0.05). As CD31 is a possible marker for both sinusoidal and vascular endothelial cells [1]; we hypothesized that the CD14^++^ cells may contain LSECs and, therefore, studied expression of other likely markers of sinusoidal endothelium on these cells. We initially subdivided the CD14^++^ population into two populations: CD14^++^CD326^+^ and CD14^++^CD326^−^. This was originally done because of an unrelated interest in CD326 as a marker of hepatocytic precursors. We isolated four populations (Fig. 1B, regions A, B, C and D) and performed expression analysis, by qPCR, for genes associated with endothelial cells. Among the four populations, only CD14^++^ cells expressed endothelial cell markers CD144 (VE-cadherin), CD202b (angiopoietin-1 or TIE-2) and CD309 (VEGF receptor 2). These cells also expressed the coagulation factors vWF and FVIII, which are produced by LSECs. There was no significant difference in expression of these five genes between the CD326^+^ and CD326^−^ subpopulations of CD14^++^ cells (*P*>0.18). Consequently, subsequent experiments did not distinguish among CD14^++^ cells based on CD326 expression.

**Figure 2:**
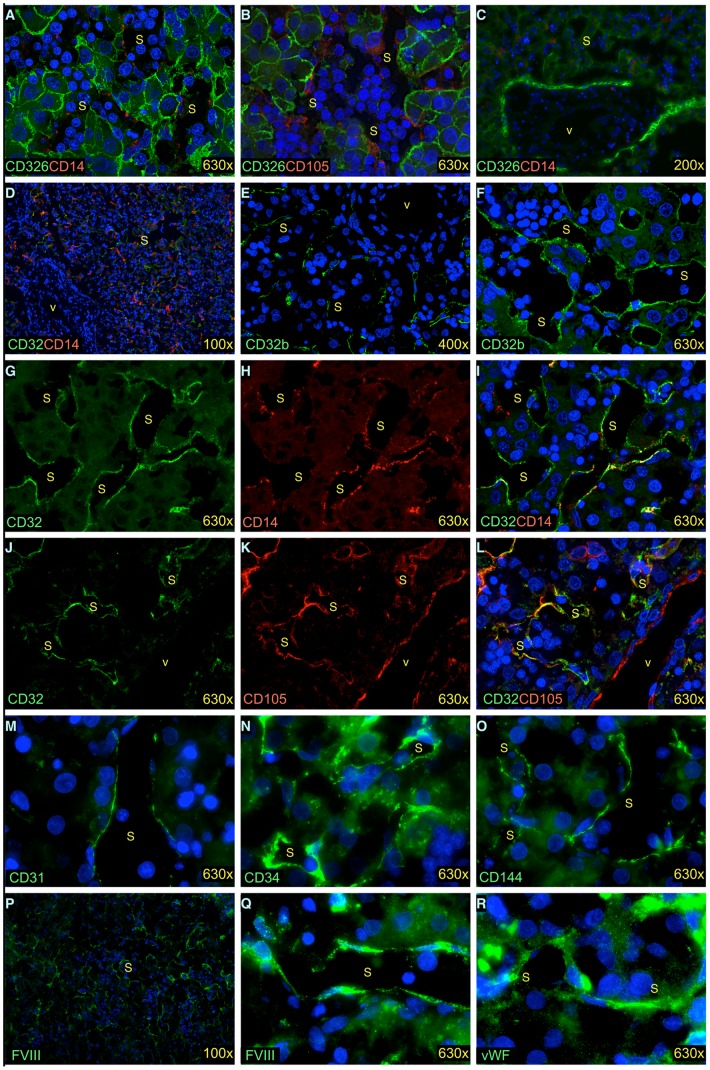
In vivo gene expression by fetal LSECs revealed by immunofluorescence staining. Fetal livers of age 9.5 weeks (A, B), 24 weeks (C–L), and 19 weeks (M–R) stained with endothelial markers. Hepatocytes stained with CD326 (A–C), nuclei stained by DAPI (blue, A–F, I, L–R). I is merged from G and H, L is merged from J and K. s-sinusoid, v-vessel with vascular endothelium.

We further analyzed expression of a number of putative LSEC and endothelial cell markers on frozen sections of human fetal livers. CD14, CD31, CD32 (Fcγ receptor II), CD32b, CD34, CD105 (endoglin), CD144, FVIII and vWF were expressed by cells lining liver sinusoids (Fig. 2). We found CD14 and CD105 expression on LSECs as early as at 9.5 weeks (Fig. 2A and 2B). CD326 staining was used to identify fetal hepatoblasts in these photomicrographs [16], [18]. A low magnification image shows, however, that CD14 expression is not observed on vascular endothelial cells found in a portal triad (Fig. 2C). Note, that portal triads in the fetal liver are bounded by a ring of hepatocytic cells that stain CD326 more brightly than parenchymal cells, whereas the inner mesenchyme and vessels do not stain with either CD326 or CD14 [16], [18].

CD32 is another antigen with a similar pattern of expression as CD14 on liver endothelial cells. A low magnification image reveals widespread staining of CD14 and CD32 in the parenchyma of a 24 weeks’ gestation liver, but neither antigen stained endothelial cells found in large vessels (Fig. 2D). The same pattern of staining was observed using polyclonal antibody that recognizes only the CD32b isoform of CD32 (Fig. 2E–F). High magnification images of CD32 and CD14 show strong co-staining on cells lining liver sinusoids (Fig. 2 G–I). Moreover, co-staining of CD32 and CD105 on cells lining liver sinusoids but not on vascular endothelial cells confirm CD32 as a specific marker of LSECs (Fig. 2 J–L).

The vascular endothelial cell markers CD31, CD34 and CD144 were confirmed to be expressed on LSECs (Fig. 2M–O). Additionally, FVIII expression was found expressed by LSECs (Fig. 2P–Q). vWF expression was found on LSECs as well as vascular endothelium (Fig. 2R and data not shown). Thus based on the antigen expression patterns observed by epifluorescence microscopy, the expression of CD14 and CD32 can be used to identify and isolate FVIII expressing LSECs.

**Figure 3:**
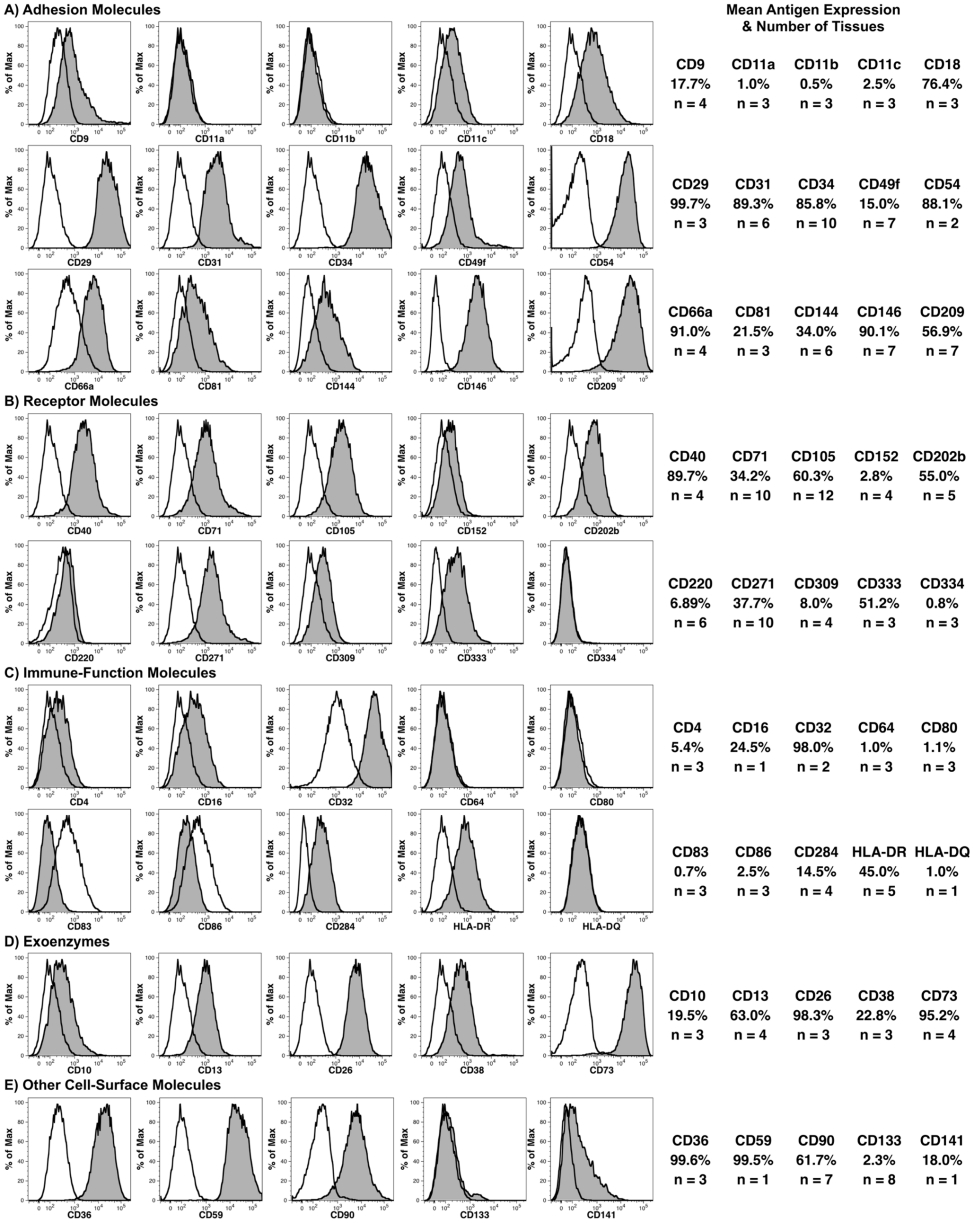
Flow cytometric analysis of antigens on LSECs. LSECs were defined as CD14^++^CD45^−^ cells using a CD45^−^ gate as shown in Fig 4A and a CD14^++^ gate as indicated by region c in Fig. 1A. Expression of the indicated antigens is shown using filled histograms, whereas staining with the corresponding isotype-control antibody is shown using an unfilled histogram. The mean frequency of positive events and the number (n) of specimens analyzed are shown on the right.

**Figure 4:**
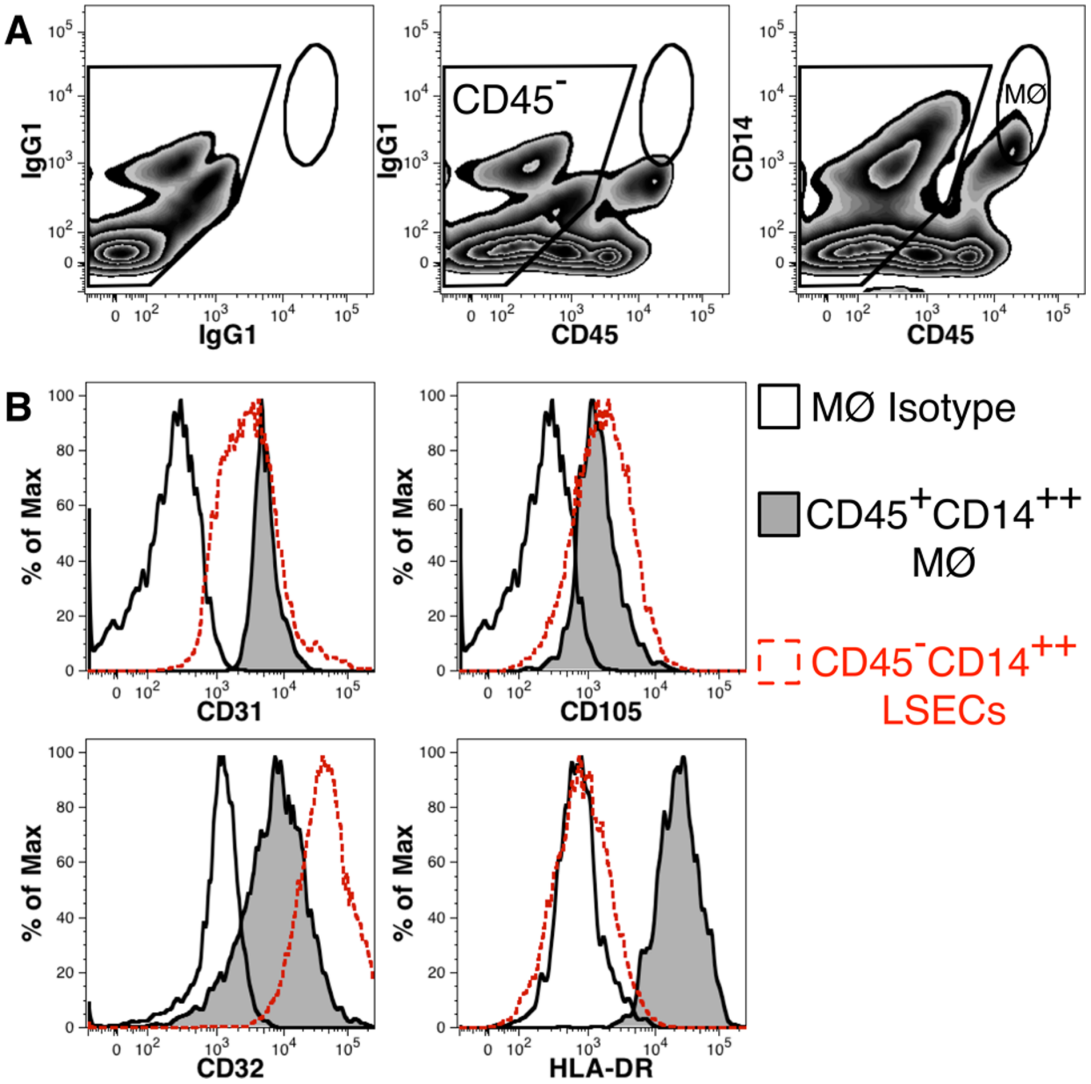
CD45 expression distinguishes LSECs from liver macrophages (MØ). Multiple populations of CD14^++^ cells are distinguished by CD45 expression (**A**). LSECs represent a population of CD14^++^ cells found among CD45^−^ cells, whereas MØ express high levels of CD45 as indicated by the oval region. Antigen expression is compared on MØ and LSECs (**B**). Background staining with isotype-control antibody is shown for the oval MØ region. The corresponding antigen expression found on LSECs is shown by gating on CD14^++^CD45^−^ cells as defined in Fig. 3.

A broader phenotypic characterization was performed on freshly isolated fetal liver cells seeking further evidence that CD14^++^ cells are LSECs (Fig. 3). For this analysis, CD14^++^ LSECs were defined as CD45^−^ cells (Fig. 4) found in region c as shown in Fig. 1A. The expression of 45 different cell-surface antigens was analyzed by flow cytometry on livers ranging in age from 13 to 23 weeks’ gestation. As indicated in Table S1, PE-conjugated monoclonal antibodies were used in almost all cases to optimize detection of the antigens.

Among the cell adhesion molecules examined (Fig. 3A), the highest expression was observed for CD29 (integrin β1), CD31, CD34, CD54 (intercellular adhesion molecule-1), CD146 (melanoma cell adhesion molecule or mucin-18) and CD209 (dendritic cell-specific intercellular adhesion molecule-3-grabbing non-integrin). Receptors expressed on CD14^++^ cells included CD40, CD71 (transferrin receptor) and CD105 (Fig. 3B). The angiopoietic growth factor receptors CD202b and CD309 were also expressed. Additionally, CD271 (low-affinity nerve growth factor receptor) and CD333 (fibroblast growth factor receptor 3) were expressed. A number of molecules associated with immune function were analyzed as well (Fig. 3C), with high levels of CD32 expression being notable. Lower levels of CD16 (Fcγ receptor III) were also observed. CD284 (toll-like receptor 4) and HLA-DR expression were also detected. Among the cell-surface enzymes analyzed (Fig. 3D), expression of CD26 and CD73 (ecto-5′-nucleotidase) were the highest, but CD10 (membrane metallo-endopeptidase), CD13 (alanine aminopeptidase) and CD38 (cyclic ADP ribose hydrolase) were also expressed at moderate levels. Lastly, high levels of CD36 (thrombospondin receptor), CD59 (protectin) and CD90 (Thy-1) were measured, and low levels CD141 (thrombomodulin) expression was observed (Fig. 3E).

LSECs share many phenotypic similarities to other parenchymal and sinusoidal cells. In particular, macrophages (Kupffer-Browicz cells) found adjacent to LSECs within the liver sinusoids have in the past been difficult to distinguish from LSECs owing to their many similarities and close association within the liver tissue. Liver macrophages were excluded from our flow cytometric analysis of LSECs based on their expression of CD45 (Fig. 4A). Macrophages express high levels of CD45 unlike LSECs. Both cell types express similar levels of CD14 (Fig. 4A) as well as the endothelial cell markers CD31 and CD105 (Fig. 4B). CD32 was also found to be expressed by macrophages, but at modestly lower levels than found on LSECs. Conversely, HLA-DR expression was notably higher on macrophages than on LSECs. Among the antigens studied, CD45 provides the clearest separation of LSECs and macrophages.

### Culture of Fetal CD14^++^ LSECs

**Figure 5:**
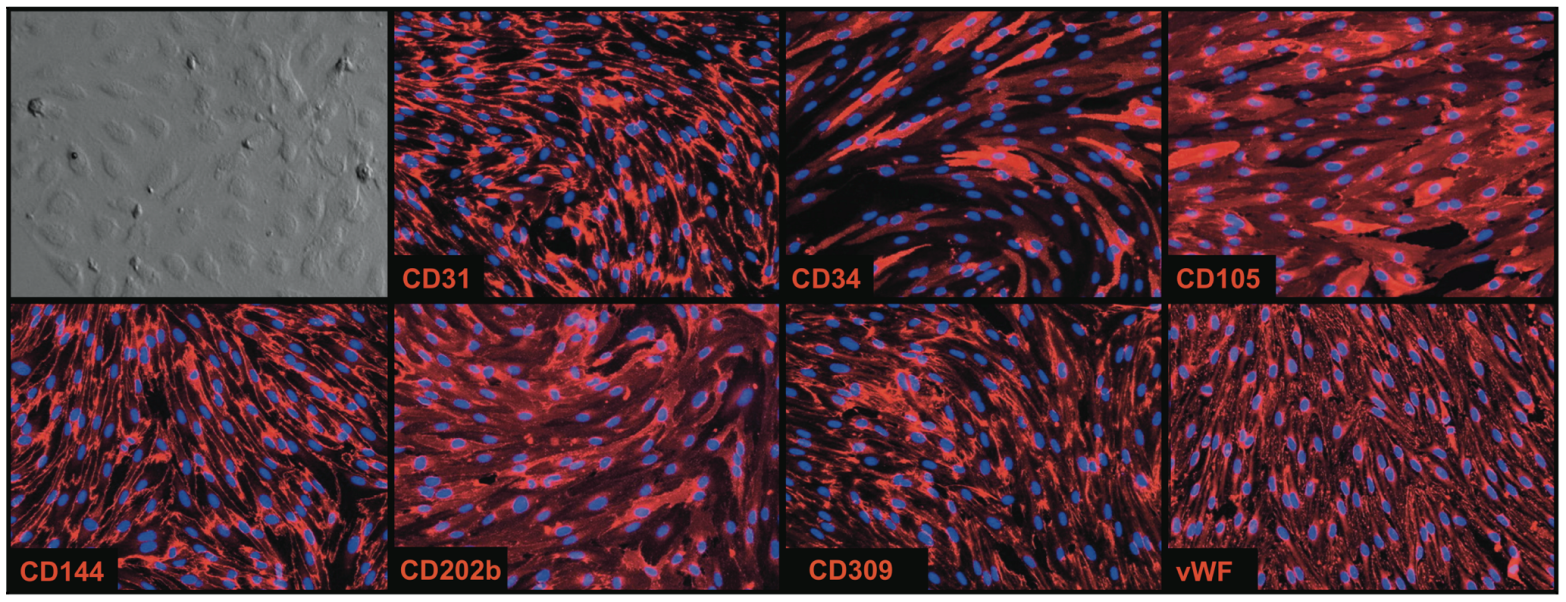
Morphology and antigen expression on cultured CD14^++^CD326^−^CD45^−^ cells. Cultured cells form a “cobble stone” monolayer and express the indicated endothelial markers (red). Nuclei stained by DAPI (blue). 200×.

To prove further that CD14^++^ cells represent LSECs, we sorted CD14^++^CD326^−^ cells and cultured them under conditions supportive of endothelial cell growth. Isolated cells formed a cobble stone layer, upon reaching confluence, which is typical for cultured endothelial cells (Fig. 5). The cultured cells also expressed CD31, CD34, CD105, CD144, CD202b, CD309 and vWF.

### Transplantation of Human Fetal Liver Cells Into uPA-NOG Mice

Suspensions of erythrocyte-depleted human fetal liver cells containing hematopoietic cells, parenchymal cells and LSECs were transplanted into uPA-NOG mice and engraftment analyzed between 100 and 233 days. Human cells were detected by flow cytometry based on expression of the pan-human marker β2 microglobulin (B2M) and absence of combination of mouse markers CD45, TER-119 and H-2k^d^.

**Figure 6:**
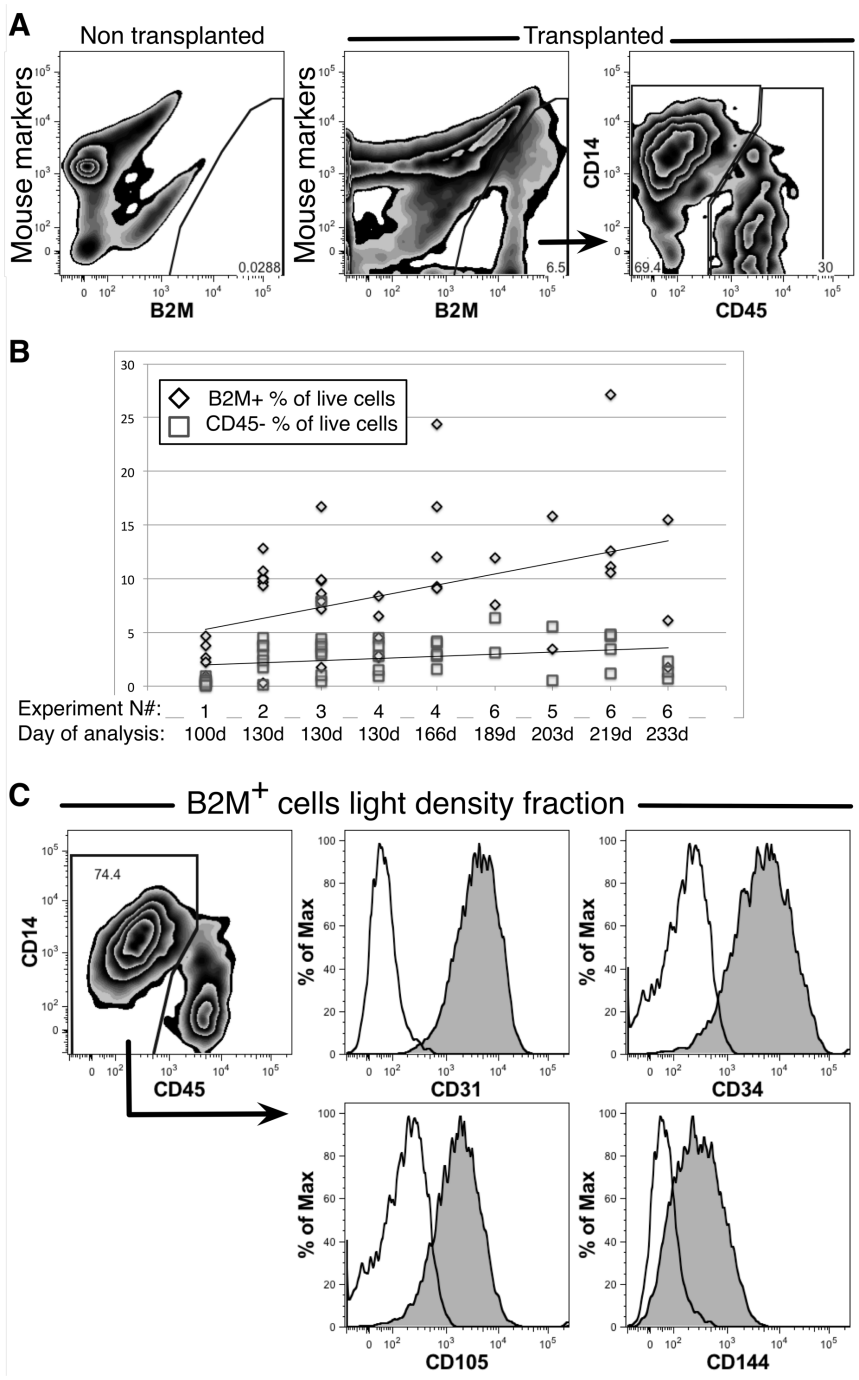
Analysis of human fetal liver cell engraftment in mouse livers. Flow cytometric analysis is shown comparing livers from untransplanted and transplanted mice. Live cells were stained with mouse markers CD45, TER-119 and H-2K^d^ and human B2M. CD14 and CD45 expression define two populations of B2M^+^ cells (**A**). A summary of 6 experiments used 44 transplanted mice shows the ratio of CD45^−^ cells among all human cells engrafted. The trend line shows a tendency of total, B2M^+^ cell, engraftment to increase with time whereas the CD45^−^ population remains more constant (**B**). Analysis of endothelial cell markers expression on the light-density fraction of transplanted mouse liver cells. Expression of the indicated antigens on CD14^++^CD45^−^ cells is shown with filled histograms and staining with isotype controls are shown as unfilled histogram (**C**).

Evidence of human hematopoietic engraftment was apparent despite the lack of cytoablative pre-conditioning before the transplants. Hematopoietic cells, representing myeloid, erythroid and lymphoid lineages, were observed in the spleens and bone marrow of uPA-NOG mice (data not shown). In the livers, two populations comprised human cells: CD45^+^ hematopoietic cells and CD45^−^ non-hematopoietic cells, the majority of which were CD14^++^ (Fig. 6A). The percentage of human cells in the liver varied in different experiments reaching up to 27% maximum of live cells and had a tendency to increase with time after transplantation. Non-hematopoietic cells repopulated mouse livers up to 8%. This frequency did not significantly increased beyond 130 days of engraftment (Fig. 6B). However, the frequency of hematopoietic cells tended to increase over time.

**Figure 7:**
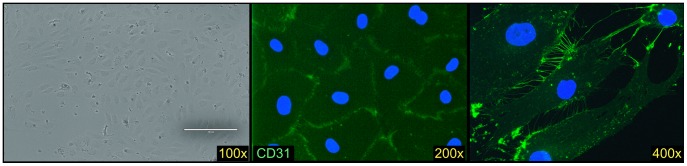
Culture of human cells recovered from the livers of transplanted uPA-NOG mice. Livers were enzymatically digested and light density cells isolated, which were cultured on collagen-coated plates with EBM2 supplied medium. Cells became confluent after 3 weeks, forming a cobble-stone layer typical for cultured human endothelial cells. The cells expressed human CD31 (green) and were negative for staining with mouse H-2Kd (not shown). Nuclei are stained with DAPI (blue).

Additional phenotypic analysis was performed on the engrafted human cells to confirm our suspicions that the non-hematopoietic cells were primarily comprised of LSECs. Light-density cells isolated from the livers of transplanted mice, enriched in CD14^++^CD45^−^ cells, were found to express CD31, CD34, CD105 and CD144 (Fig. 6C). Moreover, these cells formed a cobble-stone layer when cultured (Fig. 7), like those formed by LSECs isolated from fetal livers (Fig. 5). The cultured cells also expressed the human endothelial cell marker CD31 (Fig. 7).

**Figure 8:**
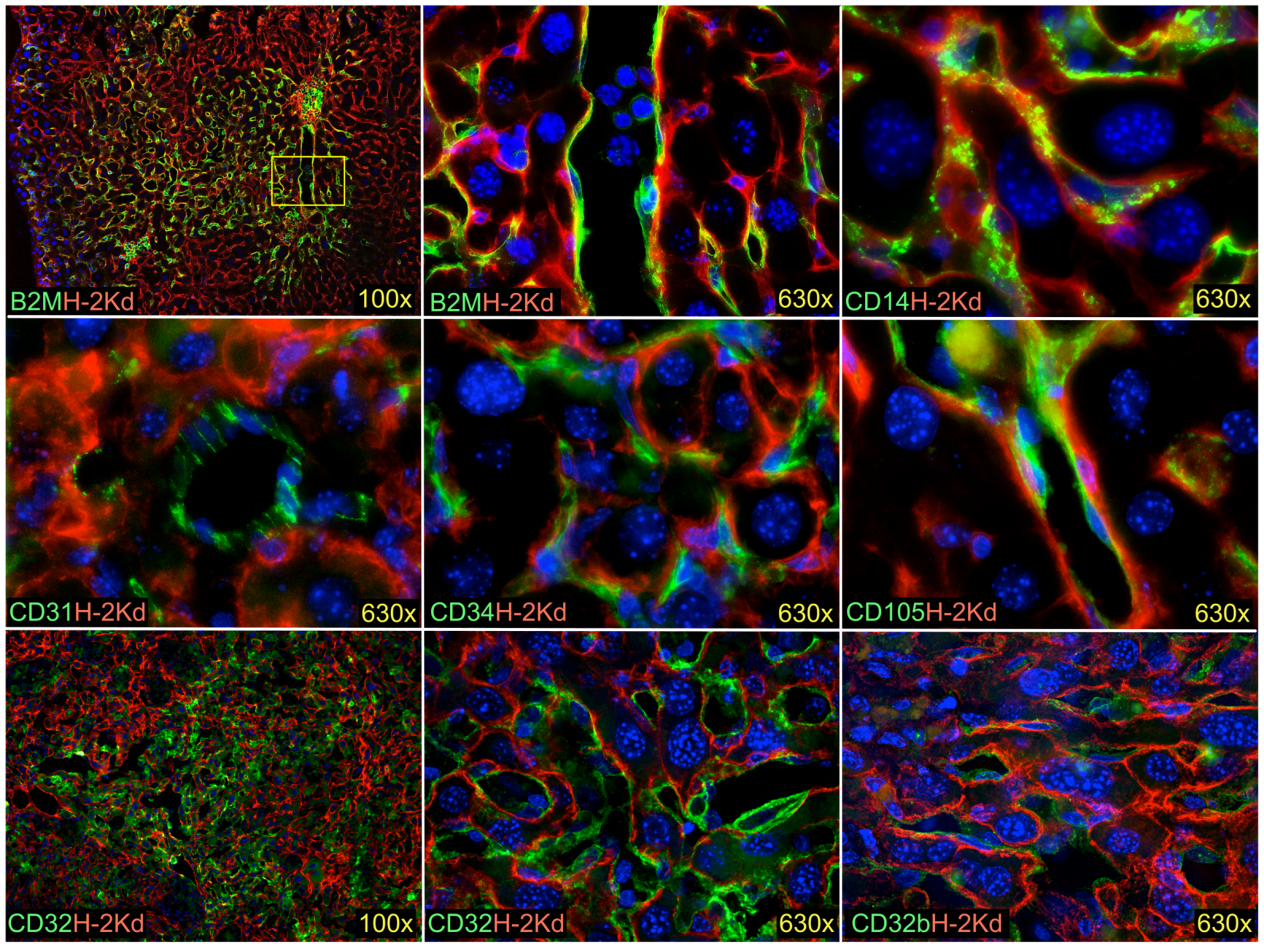
Transplanted human fetal liver cells engrafted in mouse liver. Human B2M (green) stains small elongated cells lining sinusoids between the larger mouse hepatocytes in mice transplanted with fetal human liver. Mouse cells are stained by H-2K^d^ (red). Human hematopoietic B2M^+^ cells are seen in a close-up view of a vessel. Human LSEC markers CD14, CD31, CD32, CD32b, CD34 and CD105 (green) stain small elongated cells located between mouse hepatocytes and inside sinusoids. Nuclei stained with DAPI are shown in blue.

Morphological analysis revealed that the human cells in mouse livers were relatively small, elongated with small oval nuclei and were located between mouse hepatocytes, lining sinusoids or forming small capillaries (Fig. 8). These cells expressed human CD14, CD31, CD32, CD32b, CD34 and CD105. No human hepatocytes were observed in any of the examined mouse livers transplanted with fetal liver cells.

### Human FVIII is Detected in Plasma of Transplanted Mice

**Figure 9:**
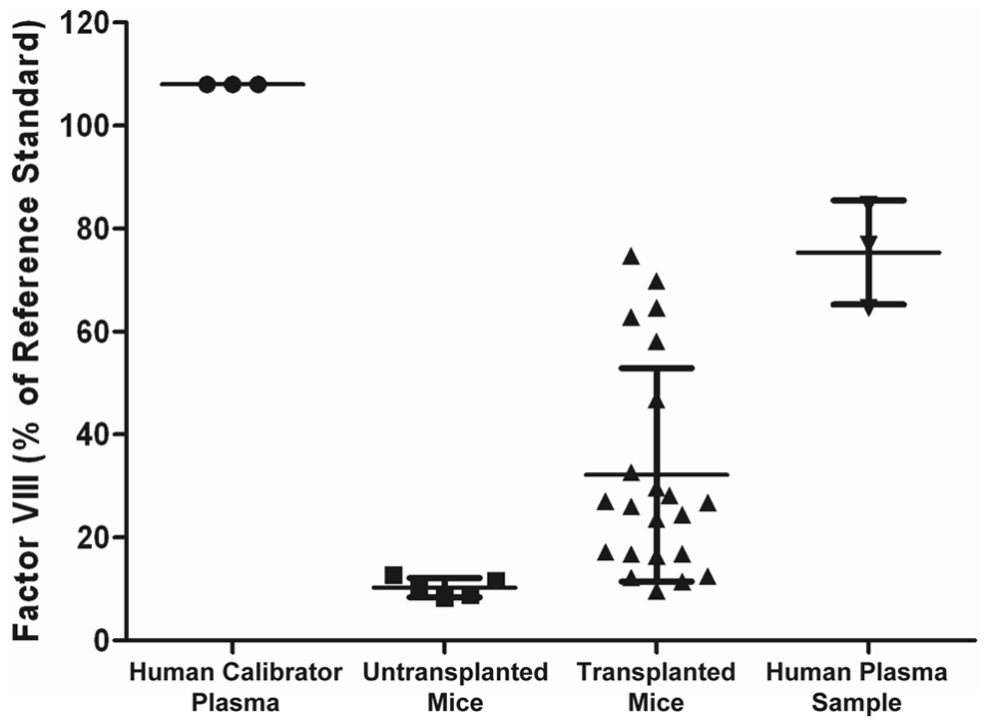
Transplanted human cells produce FVIII. The graph represents ELISA measurements of human FVIII in the plasma of untransplanted uPA-NOG mice (n = 5) and mice transplanted with human fetal liver cells (n = 22). Results are compared to a calibrated human plasma standard from the assay manufacturer and an independent human plasma sample obtained from our institute (n = 3). The calibrated plasma standard has 108% FVIII activity of a reference standard, which is equivalent to 0.95 IU/ml. Data are shown as the mean ± standard deviation.

The ability of the engrafted LSECs to produce and secrete human FVIII was measured (Fig. 9). Samples were tested from 22 transplanted and 5 untransplanted mice. The average measurements from untransplanted controls was 10% of the reference standard, ranging from 8–13%. In contrast, the average FVIII level for transplanted mice was 32%, *P*<0.01. This included 6 mice with high-levels of FVIII expression (47–75%), 8 mice with mid-levels of expression (24–33%), 4 mice with low levels of expression (17%), and 4 mice with no detectable expression (10–12%). Serial dilutions of the positive samples confirmed the presence of titratable human FVIII (not shown). An independent human plasma sample gave an average reading of 75% value of the reference standard in three experiments. These results indicated the expression of human FVIII in mice transplanted with human fetal liver cells.

### Upregulation of VEGF Expression in the Livers of uPA-NOG Mice

**Figure 10:**
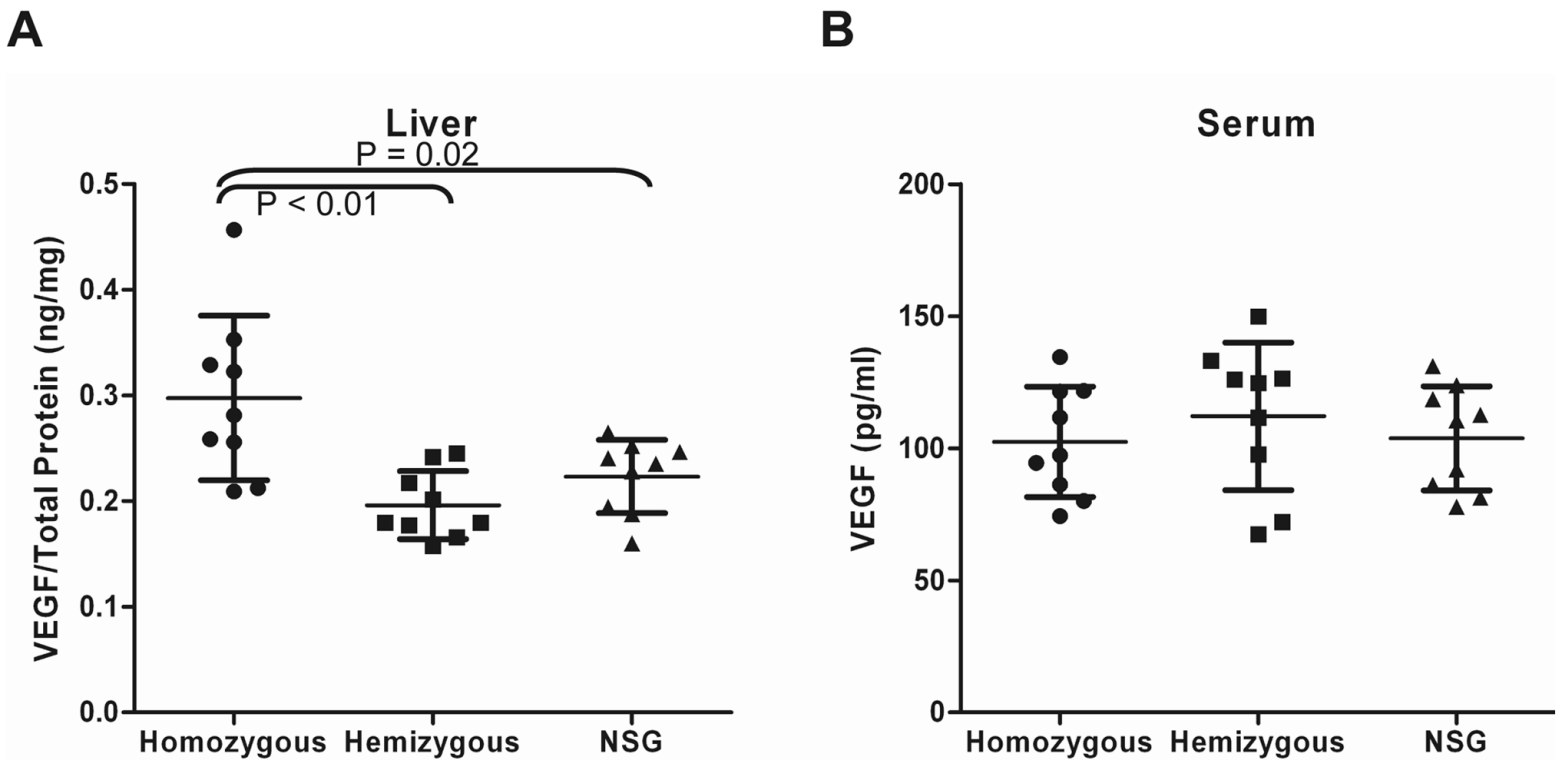
Mouse VEGF expression in livers (A) and plasma (B) of uPA-NOG homozygous, uPA-NOG hemizygous and NSG mice (n = 9 for each group). VEGF levels in the liver are shown as ratio of the amount of VEGF to total protein.

Notable engraftment of uPA-NOG livers by fetal LSECs was unexpected. Therefore, we examined more closely the liver microenvironment of mice overexpressing uPA. We did not notice any significant damage nor increased proliferation among murine LSECs (data not shown). uPA plays an important role in angiogenesis and liver regeneration [19], [20]. In particular, uPA activates matrix metalloproteases (MMPs) and increases release of growth factors such as the angiogenic factor VEGF [21]. Thus, we tested if the levels of VEGF were increased in uPA-NOG mice. We compared three groups of mice: uPA-NOG homozygous, uPA-NOG hemizygous, and NSG – immunodeficient mice with no uPA transgene. Each group contained 9 mice of different ages and sexes in the same proportions. The average level of VEGF in the livers of homozygous mice was 0.3 ng/mg, which was 50% higher than in hemizygous or NSG mice (Fig. 10A). No differences were observed between hemizygous and NSG mice. No correlations were apparent between age or sex and the levels of VEGF. Plasma VEGF levels were very low and did not show any difference among the tested groups of animals (Fig. 10B).

### Adult Liver Cells Transplanted into uPA-NOG Mice

**Figure 11:**
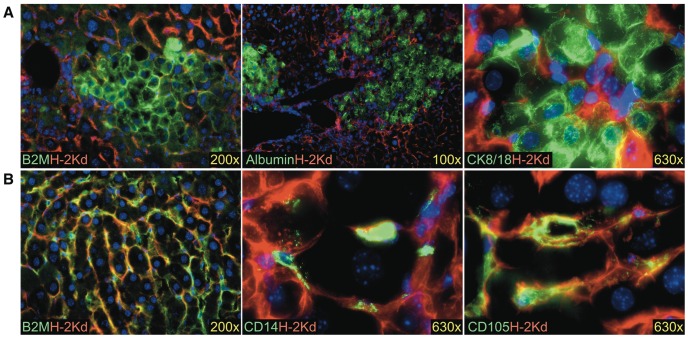
Transplantation of adult human liver cells results in hepatocyte and LSEC engraftment. Examples of hepatocytes are shown in (**A**) and LSECs in (**B**). Mouse H-2K^d^ is shown in red; human markers are shown in green. Nuclei are stained with DAPI (blue).

Since no hepatocyte engraftment was detected in 9 experiments using fetal donor cells, we choose to confirm hepatocyte engraftment of these mice with adult hepatocytes. Adult liver cells were able to engraft mouse parenchyma (Fig. 11A). Indeed, most of the engrafted cells from the adult cell transplants appeared to be hepatocytes. These were large polygonal cells expressing human hepatocyte markers albumin and cytokeratin 8/18 (CK8/18). No albumin expression was ever detected in mice transplanted with fetal cells. Although most of the adult graft was comprised of hepatocytes, apparently, there was also a fraction of non-parenchymal cells. Immunostaining of transplanted mouse livers for B2M revealed several colonies of human cells with morphology similar to the LSECs from fetal grafts (Fig. 11B). These cells were positive for CD14 and CD105. Thus, adult and fetal LSECs can successfully engraft the livers of uPA-NOG mice.

## Discussion

Successful transplantation of human liver cells into uPA-NOG mice was demonstrated resulting in engraftment of LSECs and production of human FVIII. We present an extensive phenotypic profile of fetal LSECs, which should prove valuable in future efforts in the identification, isolation and study of LSECs. Freshly isolated and transplanted LSECs can be reliably identified based on their expression of CD14 and CD32 as well as a lack of CD45 staining to exclude hematopoietic cells such as macrophages. Furthermore, we demonstrated that whereas fetal and adult LSECs engrafted uPA-NOG mice, only adult hepatocytes could engraft uPA-NOG mice.

Despite numerous studies describing LSECs, their molecular identity remains a controversial issue [1]. We sought to provide a thorough phenotypic description of freshly-isolated fetal LSECs to better understand the differences between these cells and other endothelial cells. We observed a sizable population of cells expressing mid to high levels of CD14 (CD14^++^ cells) in human fetal livers, which were neither hematopoietic nor parenchymal cells. Previously, expression of CD14 was described on human fetal LSECs after 20 weeks of gestation [6], in adult human liver sections [22], and on rat LSECs during endotoxemia [23]. We also observed CD14 expression on LSECs in tissue sections as young as 9.5 weeks’ gestation and by flow cytometry on 13 weeks’ gestation livers. CD14 is a receptor for lipopolysaccharide, which together bind CD284. Our results demonstrate constitutive expression of these molecules prior to any exposure of the LSECs to lipopolysaccharide from the gut, as was also suggested for murine LSECs [24].

Flow cytometric analysis revealed expression of endothelial cell markers such as CD31, CD34 and CD105 on LSECs. CD32 expression has been shown on LSECs, but not vascular endothelial cells [25], and we show high levels of CD32 expression, as well as the CD32b isoform, on fetal LSECs. We also did not observe expression of CD32 on vascular endothelial cells in the liver. LSECs are also known to express a number of molecules associated with antigen presentation [1], [10]. We observed expression of CD4, CD11c, CD40, CD54 and HLA-DR, but we did not detect any expression of the T-cell co-activation ligands CD80 and CD86, in contrast to what has been reported for murine LSECs [10]. In mice, LSECs have been shown to promote T-cell tolerance, thus these cells play an important role in allogeneic liver transplantation [26]–[29]. The tolerizing properties of LSECs may allow for greater mismatch in major histocompatibility antigens when considering allogeneic LSEC transplantation as a therapy for hemophilia A.

Different animal models have been used to study LSEC transplantation. For instance, DPPIV deficient rats were shown to incorporate DPPIV^+^ rat LSECs [12], [13]. Also, transplantation of murine LSECs could prevent hemophilia in FVIII knockout mice [9], [14]. In some experiments livers of recipient animals were preconditioned by irradiation [13] or chemically [9], [12] in order to disrupt the endogenous sinusoidal layers. In the present study uPA-NOG mice were used without preconditioning. No defects in LSECs were apparent in these mice, yet they provided conditions beneficial for engraftment of human LSECs. We hypothesized that overexpression of uPA, an important component of extracellular matrix network in the liver of these mice, aids in creating a permissive microenviroment for LSEC engraftment. A role of uPA has been shown in liver repair and remodeling [30]. uPA activates MMPs which in turn trigger VEGF signaling [21]. VEGF cleavage by MMPs [31], plasmin [32], or directly by uPA [33] is required for its mitogenic function. Furthermore, uPA/uPA receptor system is required for VEGF induced cell migration [19]. Additionally, uPA gene therapy of liver cirrhosis has been observed to result in upregulation of VEGF, promoting liver regeneration [20]. VEGF stimulates liver regeneration by specifically recruiting LSECs [34], [35]. Conversely, blocking of VEGF in injured livers prevents migration of LSEC precursors from the bone marrow [36]. uPA-NOG mice have increased level of VEGF in the livers, suggesting that overexpression of uPA is resulting not only in an impaired liver parenchyma but also providing the angiogenic signaling required for successful LSEC engraftment.

We confirmed the observation that the uPA-NOG mouse is a permissive host for adult human hepatocytes [15]. However, we observed no definitive evidence of fetal hepatocyte engraftment, in line with a prior study demonstrating superior engraftment with adult hepatocytes compared to fetal cells [37]. Others have reported engraftment of fetal-liver derived hepatocytes in mice [18], [38]. There are notable differences between fetal and adult parenchymal cells, but which of these differences account for the poor engraftment of fetal cells in the adult liver environment are still unknown [16], [18].

Demonstration of successful human LSEC transplantation and production of FVIII offers hope for the development of a cellular therapy for hemophilia A. Hemophilia A treatment currently requires lifelong administration of plasma-derived or recombinant FVIII. This treatment, although lifesaving, is associated with immune reactions, unending medical care and considerable cost [39]. Thus, about 30% of children receiving recombinant or plasma derived FVIII infusions developed inhibitory antibodies to it [40]. Successful transplantation of a sufficient number of LSECs capable of prolonged, or even life-long, FVIII production would be a significant advancement for the treatment of hemophilia A.

We demonstrate that LSECs are the only source of FVIII production in the fetal human liver and that these cells continue to secret FVIII after transplantation into mice. Therefore we suggest that fetal LSECs could be a potential cell source for human transplantation for treatment of hemophilia A. Fetal liver has been used as a source for human transplantation before [41], [42]. Nonetheless, the number of LSECs that can be harvested from fetal tissue is limited by their size and gestation age. If fetal LSECs could be successfully expanded ex vivo while maintaining their functional properties after transplantation, then sufficient donor cells may be obtained. Successful cellular therapy for hemophilia A will depend on a number of factors including the number of cells in the graft, the frequency of transplants, the ability of the transplanted cells to engraft, the capacity of the engrafted cells to grow and their survival in a foreign host. Alternatively, the technology to produce patient-specific induced pluripotent stem cells that can be genetically corrected and then expanded and differentiated into LSECs offers another possible treatment for Hemophilia A. Such a therapy would also depend on adequate cell culture systems to produce a graft of sufficient size as well as development of robust and safe technologies for the creation of pluripotent stem cells and performing the required genetic therapy on these cells. A deeper understanding of the embryonic and fetal development of LSECs and use of the uPA-NOG mouse model can aid in the development of such therapies.

## Supporting Information

Table S1 **Antibody reagents and their sources used in the study.**
(PDF)

Table S2 **Quantitative polymerase chain reaction primer sequences used in the study.**
(PDF)
