# Supplementary material for: Production of Factor VIII by Human Liver Sinusoidal Endothelial Cells Transplanted in Immunodeficient uPA Mice
Source: PLoS One. 2013 Oct 22;8(10):e77255. doi: 10.1371/journal.pone.0077255 (PMC3805584; doi:10.1371/journal.pone.0077255)
Supplement: Table S1 — Antibody reagents and their sources used in the study. (PDF) [file pone.0077255.s001.pdf]

Table S1. **Antibody reagents and their sources used in the study.**

Antibody	Fluorophore	Company	Catalog No.	Clone	Isotype
Antibodies recognizing human antigens					
CD3	PE-Cy7	BioLegend	300420	UCHT1	IgG1
CD4	PE	BD Biosciences	550630	IV T114	IgG1
CD7	PE	BioLegend	343106	CD7-6B7	IgG2a
CD9	PE	BD Biosciences	555372	M-L13	IgG1
CD10	PE	Life Technology	MHCD1004	5-1B4	IgG1
CD11a	PE	Life Technology	MHCD11a04	MEM-25	IgG1
CD11b	PE	Life Technology	MHCD11b04	CR3 (Bear-1)	IgG1
CD11c	PE	BD Biosciences	347637	S-HCL-3	IgG2b
CD14	unconjugated	BioLegend	325602	HCD14	IgG1
CD14	APC	BioLegend	325-608	HCD14	IgG1
CD14	FITC	BioLegend	325604	HCD14	IgG1
CD14	FITC	Life Technology	MHCD1401	Tük4	IgG2a
CD14	PE-Cy7	BioLegend	325618	HCD14	IgG1
CD16	PE	Beckman Coulter	IM1238	3G8	IgG1
CD18	PE	Life Technology	MHCD1804	MEM-48	IgG1
CD19	PE	BioLegend	302208	HIB19	IgG1
CD26	PE	BioLegend	302706	BA5b	IgG2a
CD29	PE	BD Biosciences	55543	MAR4	IgG1
CD31	AF488	BioLegend	303110	WM59	IgG1
CD31	PE	Life Technology	MHCD3104	MBC 78.2	IgG1
CD32	PE	BioLegend	303206	FUN-2	IgG2b
CD32b	unconjugated	Abcam	ab110076	polyclonal	
CD34	APC	BioLegend	343510	581	IgG1
CD34	FITC	BD Biosciences	555821	581	IgG1
CD34	PE	Life Technology	CD34-581	581	IgG1
CD34	PE-Cy7	BioLegend	343516	581	IgG1
CD36	FITC	Beckman Coulter	IM0766	FA6.152	IgG1
CD38	PE	BD Biosciences	347687	HB7	IgG1
CD40	PE	Beckman Coulter	IM1936	mAb89	IgG1
CD45	FITC	BioLegend	304006	H130	IgG1
CD45	PE	BioLegend	304008	H130	IgG1
CD45	PE-Cy7	BioLegend	304016	H130	IgG1
CD49f	PE	BioLegend	313612	GoH3	IgG2a
CD54	PE	BioLegend	322708	HCD54	IgG1
CD56	APC	BioLegend	318310	HCD56	IgG1
CD59	PE	Life Technology	MHCD5904	MEM-43	IgG2a
CD64	PE	Life Technology	MHCD6404	32.2	IgG1
CD66a	PE	R&D Systems	FAB2244P	283340	IgG2b
CD71	PE	BD Biosciences	555537	L01.1	IgG2a
CD71	PE	Beckman Coulter	IM2001	YDJ1.2.2	IgG1
CD73	PE	BD Biosciences	555749	MOPC-21	IgG1
CD80	PE	Beckman Coulter	IM1976	MAB104	IgG1
CD81	PE	Beckman Coulter	IM2579	JS64	IgG2a
CD83	PE	Beckman Coulter	IM2218U	HB15a	IgG2b
CD86	PE	Beckman Coulter	IM2729	HA5.2B7	IgG2b
CD90	PE	BD Biosciences	555596	5E 10	IgG1
CD105	unconjugated	Life Technology	MHCD10500	SN6	IgG1
CD105	PE	Life Technology	MHCD10504	SN6	IgG1
CD105	APC	BioLegend	323208	43A3	IgG1
CD133	PE	Miltenyi Biotec	130-080-801	AC133	IgG1
CD141	PE	BD Biosciences	559781	1A4	IgG1
CD144	PE	R&D Systems	FAB9381P	123413	IgG2b
CD144	FITC	BD Biosciences	55-7H1	560411	IgG1
CD146	PE	R&D Systems	FAB932P	128018	IgG1
CD152	PE	BD Biosciences	555853	BNI3	IgG2a
CD202b	PE	R&D Systems	FAB3131P	83715	IgG1
CD203c	PE	BioLegend	324606	NP4D6	IgG1
CD203c	APC	Miltenyi Biotec	130-092-344	FR3-16A11	IgG1
CD220	PE	BD Biosciences	559955	3E6/IR	IgG1
CD209	PE	BioLegend	330105	9E9A8	IgG2a
CD235a	FITC	Life Technology	MHGLA014	CLB-ery-1	IgG1
CD271	PE	BD Biosciences	557196	C40-1457	IgG1
CD284	PE	BioLegend	312806	HTA125	IgG2a
CD309	PE	R&D Systems	FAB357P	89106	IgG1
CD326	APC	Miltenyi Biotec	130-091-254	HEA-125	IgG1
CD333	PE	R&D Systems	FAB766P	136334	IgG1
CD334	PE	BioLegend	324306	4FR6D3	IgG1
Albumin	FITC	EY laboratories	FA-2113-2	polyclonal	
β-2 microglobulin	FITC	BioLegend	316304	2M2	IgG1
CK8/18	unconjugated	Life Technology	18-0213	Zym5.2	IgG1
FVIII	unconjugated	Affinity Biologicals Inc.	F9C-EIA-C	polyclonal	
HLA-DR	PE	BD Biosciences	555561	TÜ36	IgG2b
HLA-DQ	FITC	BD Biosciences	347453	SK10	IgG1
HLA-ABC	APC-Cy7	BioLegend	311426	W6/32	IgG2a
vWF	unconjugated	Beckman Coulter	PN IM0150	4F9	IgG1
Non-specific antibodies for isotype control					
IgG1	APC	BioLegend	400120	MOPC-21	
IgG1	FITC	BioLegend	400108	MOPC-21	
IgG1	PE	BioLegend	400114	MOPC-21	
IgG1	PE-Cy7	BioLegend	400126	MOPC-21	
IgG2a	APC	BioLegend	400220	MOPC-173	
IgG2a	FITC	BioLegend	400208	MOPC-173	
IgG2a	PE	BD Biosciences	559319	G155-178	
IgG2b	PE	BD Biosciences	555743	27-35	
Antibodies recognizing mouse antigens					
CD45	Pacific Blue	BioLegend	103126	30-F11	
H2-K^d	Pacific Blue	BioLegend	116616	SF1-1.1	IgG2a
H2-K^d	AF647	BioLegend	116612	SF1-1.1	IgG2a
TER-119	Pacific Blue	BioLegend	116232	TER-119	
Secondary antibodies					
IgG1 goat anti-mouse	AF488	Life Technology	A21121		
IgG1 goat anti-mouse	AF568	Life Technology	A21124		
IgG2a goat anti-mouse	AF568	Life Technology	A21134		
IgG2b goat anti-mouse	AF568	Life Technology	A21144		
donkey anti-sheep	AF488	Life Technology	A11015		
rabbit anti-goat	FITC	Sigma Immuno Chemicals	F-2016		
