# Supplementary material for: Production of Factor VIII by Human Liver Sinusoidal Endothelial Cells Transplanted in Immunodeficient uPA Mice
Source: PLoS One. 2013 Oct 22;8(10):e77255. doi: 10.1371/journal.pone.0077255 (PMC3805584; doi:10.1371/journal.pone.0077255)
Supplement: Table S2 — Quantitative polymerase chain reaction primer sequences used in the study. (PDF) [file pone.0077255.s002.pdf]

Table S2. Quantitative polymerase chain reaction primer sequences used in the study.

Gene	Primers	Position	GenBank accession no.	Product size (bp)	References
18S rRNA (F)<br>18S rRNA (R)	5'- TTCGGAACTGAGGCCATGAT<br>5'- CGAACCTCCGACTTTCGTTT	1842–1861<br>1992–1973	gi22760900	151	[1]
CD31 (F)<br>CD31 (R)	5'- CCTGATGCCGTGGAAAGC<br>5'- TCCAGGGATGTGCATCTGG	2374–2391<br>2458–2440	gi110347450	85	[2]
CD144 (F)<br>CD144 (R)	5'- GCCATCGATAATTCTGGACG<br>5'- CTTCCACCACGATCTCATAC	662-681<br>746-722	gi109734344	85	[2]
CD202b (F)<br>CD202b (R)	5'- GAGGAGCGAAAGACCTACG<br>5'- GCAGAACAGTCAATTCCTGC	3592-3610<br>3662-3643	gi88758595	70	[2]
CD309 (F)<br>CD309 (R)	5'- CAGACGGACAGTGGTATGG<br>5'- AGTGATATCCGGACTGGTAG	3831-3849<br>3993-3974	gi124297527	165	[2]
Factor VIII (F)<br>Factor VIII (R)	5'- GGCTTCCCATCCTGTCAGTC<br>5'- GGGTCAGAGGCCATTGGACC	501-520<br>680-661	gi192448441	180	
vWF (F)<br>vWF (R)	5'- AGGAAGACCACCTGCAACC<br>5'- GCCGTAGGCAAACATCTCC	8106-8126<br>8194-8175	gi89191867	89	[2]

F, forward; R, reverse.

Supplementary References

1. Lie-A-Ling M, CT Bakker, T Deurholt, et al. (2006). Selection of tumour specific promoters for adenoviral gene therapy of cholangiocarcinoma. J Hepatol 44:126–133.
2. Timmermans F, F Van Hauwermeiren, M De Smedt, et al. (2007). Endothelial outgrowth cells are not derived from CD133þ cells or CD45þ hematopoietic precursors. Arterioscler Thromb Vasc Biol 27:1572–1579.
